# Molecular detection and hematological alterations in dogs infected by *Babesia vogeli* from Paraguay

**DOI:** 10.29374/2527-2179.bjvm008824

**Published:** 2025-04-22

**Authors:** Liz Aurora Castro Rojas, Elvio Gayozo, Guadalupe Gómez, Pedro Torres, Deydra Valenzuela Zaracho, Raquel Pedrozo Prieto

**Affiliations:** 1 Departamento de Genética y Zootecnia, Facultad de Ciencias Veterinarias, Universidad Nacional de Asunción, San Lorenzo, Central, Paraguay.; 2 Departamento de Biología, Facultad de Ciencias Exactas y Naturales, Universidad Nacional de Asunción, San Lorenzo, Central, Paraguay.; 3 Departamento de Clínicas Veterinarias, División Patología Clínica, Facultad de Ciencias Veterinarias, Universidad Nacional de Asunción, San Lorenzo, Central, Paraguay.; 4 Departamento de Clínicas Veterinarias, División Hospital Veterinario, Universidad Nacional de Asunción, Facultad de Ciencias Veterinarias, San Lorenzo, Central, Paraguay.; 5 Carrera de Ciencias Veterinarias, Facultad de Ciencias Veterinarias, Universidad Nacional de Asunción, San Lorenzo, Central, Paraguay.; 6 Sistema Nacional de Investigadores (SISNI-CONACYT), Asunción, Central, Paraguay.

**Keywords:** canine babesiosis, hematology, Vector-Borne Pathogens, PCR, babesiose canina, hematologia, Patógenos Transmitidos por Vetores, PCR

## Abstract

Babesiosis is a vector-borne disease caused by an intraerythrocytic protozoan parasite of the genus *Babesia*. There are several species of *Babesia* that can infect dogs, however, *B. vogeli* is the most widely distributed in South America. Anemia and thrombocytopenia are the most frequent hematologic alterations reported in dogs with babesiosis. The aim of the present study was to perform a molecular characterization of the *Babesia* species and describe the hematological findings regarding dogs with the *Babesia* infection. Blood samples were collected from 35 dogs living in an urban area with symptoms compatible with babesiosis, and the data was analyzed using molecular as well as hematological approaches. Molecular detection was performed by nested Polymerase Chain Reaction (PCR) targeting the 18S rRNA gene. To identify species, positives samples were sequenced and analyzed. Among the total number of samples, four (11%) were reported as positive due to a high identity (99-100%) and were clustered with the *B. vogeli* clade. The main hematological alteration found in infected dogs was thrombocytopenia (100%). Other abnormalities were also observed, although to a lesser extent, such as: normocytic normochromic anemia, monocytosis and eosinopenia. It is important to emphasize that this research is the first study involving molecular detection and hematology abnormalities in dogs with *B. vogeli* from Paraguay.

## Resumo

A babesiose é uma doença transmitida por protozoários intraeritrocíticos do gênero *Babesia.* Há várias espécies de *Babesia* que infectam cães, mas a *B. vogeli* é a mais amplamente distribuída na América do Sul. A anemia e a trombocitopenia são as alterações hematológicas mais frequentemente relatadas em cães com babesiose. O objetivo do presente estudo foi caracterizar molecularmente as espécies de *Babesia,* descrevendo os achados hematológicos de cães com infecção por *Babesia*. Amostras de sangue foram coletadas de 35 cães que viviam em uma área urbana com sinais compatíveis com babesiose, sendo analisadas por meio de abordagens moleculares e hematológicas. A detecção molecular foi realizada utilizando a reação em cadeia da polimerase aninhada (PCR - *Polymerase Chain Reaction*) visando o gene 18S rRNA. Para identificar as espécies, as amostras positivas foram sequenciadas e analisadas. Entre o número total de amostras, quatro (11%) foram registradas como positivas devido a uma alta identidade (99-100%) e foram agrupadas no clado *Babesia vogeli*. A principal alteração hematológica encontrada em cães infectados foi a trombocitopenia (100%). Outras anormalidades também foram observadas, embora em menor grau, tais como: anemia normocítica normocrômica, monocitose e eosinopenia. É importante ressaltar que esta pesquisa é o primeiro estudo envolvendo detecção molecular e anormalidades hematológicas em cães com *B. vogeli* do Paraguai.

## Introduction

Babesiosis is a hemolytic disease caused by a protozoan of the genus *Babesia* (Moraes et al., 2014). They are globally distributed tick-borne parasites that infect the red blood cells of a wide range of vertebrate hosts, including humans (Annoscia et al., 2017).

Domestic dogs are infected with several *Babesia* sp. that cause severe diseases. These include: *Babesia rossi*, *Babesia canis*, *Babesia vogeli*, *Babesia gibsoni*, *Babesia conradae*, *Babesia vulpes*, *Babesia coco* (unofficial name) and *Babesia negevi* (Baneth et al., 2020; Zygner et al., 2023). *B. canis, B. vogeli, Babesia rossi* and *B. coco* are considered large piroplasms due to the fact that in their developmental stages, such as trophozoites and merozoites, they are bigger than those in the second group of Babesia, *i.e*., the small *Babesia* sp., which includes *B. gibsoni, B. conradae,* and *B. vulpes* (Köster et al., 2015). On that note, the developmental stages of *B. negevi* are smaller than large babesiae, but larger than the small *Babesia* species (Baneth et al., 2020).

*Babesia*-infected dogs can be asymptomatic (Buddhachat et al., 2012) or present various clinical signs that may range from mild to severe, acute and potentially fatal (Rawangchue & Sungpradit, 2020). Clinical manifestations differ according to the *Babesia* species and strains, as well as to their virulence and individual factors which depend on their host’s conditions, namely: age, status of the immune system, and the presence of other infections (Birkenheuer et al., 2004; Irwin, 2009; Jacobson, 2006). Frequent clinical signs associated with canine babesiosis are apathy, weakness, anorexia, pale mucous membranes, fever, swollen lymph nodes, enlarged spleen, jaundice and pigmenturia (Solano-Gallego et al., 2016). A complete blood count usually presents many abnormalities, such as hemolytic anemia, leucopenia, neutropenia, thrombocytopenia and lymphopenia (Kostro et al., 2015; Petra et al., 2018).

The diagnosis of *Babesia* infection in dogs is carried out through several diagnostic techniques, such as the cytological stained blood smear examination, the serological test and the molecular analysis (Panti-May & Rodríguez-Vivas, 2020). However, the cytological diagnosis of babesiosis is often hampered by the transient presence of the parasites in the peripheral blood, as well as by their pleomorphic nature (Annoscia et al., 2017). In addition, the sensitivity of this method is lower than molecular diagnostics and cannot provide a positive diagnosis with absolute certainty (Solano-Gallego et al., 2016). Serological tests, on the other hand, are more effective due to the increasing possibility to detect the infection; nevertheless, cross-reactivity between the *Babesia* sp. and other protozoan parasites may occur (Solano-Gallego & Baneth, 2011; Zanette et al., 2014). Alternately, molecular techniques like PCR, qPCR and PCR-RFLP provide a highly sensitive and specific detection of *Babesia* in blood and other tissues. These methods are useful for screening dogs with low parasitemia or clinical cases with signs and/or hematological alterations suspicious of tick-borne infection (Solano-Gallego & Baneth, 2011). In this context, several investigations have been conducted using the PCR amplification of the partial 18S rRNA gene, because it allows the identification of different *Babesia* sp. (Assad et al., 2020; Birkenheuer et al., 2004; Duh et al., 2004; Inácio et al., 2019; Matjila et al., 2004; Sá et al., 2006).

Given the relevance of such parasite to canine health as well as the scarcity information on this subject in Paraguay, the present study aimed to molecularly characterize the species of *Babesia* and describe the hematological changes in dogs presenting *Babesia* infection treated at the Hospital Veterinary of the Facultad de Ciencias Veterinarias, from the Universidad Nacional de Asunción, Paraguay.

## Materials and methods

### Ethics issues

The samples used in this study consisted of blood draws collected for routine diagnosis from dogs attending the veterinary hospital. Therefore, no formal ethical approval was required.

### Sampling

Animals were included in the investigation with the informed consent of their owners. The medical history and physical examination findings were extracted from the medical records of all dogs. The type of sampling was non-probabilistic by convenience. Three milliliters of blood were collected and transferred to EDTA tubes by performing a venipuncture of the cephalic vein in 35 dogs with different ages, sex and breeds that lived in urban areas of the Central region, that were patients at the Veterinary Hospital of the Facultad de Ciencias Veterinarias from August to December 2018. Animals with clinical suspicion of canine babesiosis and/or infestation with ticks were included in this research. The criteria for selection was the presence of two or more clinical signs, namely: apathy, weakness, anorexia, pale mucous membranes, fever, swollen lymph nodes, jaundice and/or pigmenturia (Solano-Gallego et al., 2016). Animals that had already received previous treatment for any hemoparasitosis, as well as those presenting samples that were positive for other hemoparasites by microscopy observation were excluded from the present study.

### DNA extraction

For each sample, the total genomic DNA was extracted from 100 µL blood collected in EDTA tubes using a PureLink^TM^ Genomic DNA Mini Kit (Invitrogen^®^, USA), in accordance with the manufacturer’s recommendations. The DNA samples were subsequently stored at -20°C until further use.

### PCR amplification

A nested PCR assay was conducted to amplify a partial region of the piroplasmids’ 18S rRNA gene. The first PCR consisted of primers BTF1 (5’- GGCTCATTACAACAGTTATAG -3’) and BTR1 (5’- CCCAAAGACTTTGATTTCTCTC -3’) which amplified an approximately 930 bp long fragment; the second PCR consisted of primers BTF2 (5’ CCGTGCTAATTGTAGGGCTAATAC - 3’) and BTR2 (5’- GGACTACGACGGTATCTGATCG - 3’), which amplified an approximately 800 bp long fragment (Jefferies et al., 2007).

The PCR reaction was carried out in a final volume of 25 µL PCR, containing 1X buffer PCR, 1.5mM of MgCl_2_, 0.2 mM of dNTPs, 0.2 µM of each primer, 0.6875 U/µL of Taq polymerase (Qiagen, Hilden, Germany) and 5 µL of DNA template. Amplification was performed on a C1000™ thermal cycler (BIO-RAD, Singapore). The primary conditions of amplification consisted in an initial denaturation at 94°C for 3 min, followed by 45 cycles of denaturation at 94°C for 30 s, annealing at 58°C for 30 s and extension at 72°C for 30 s, followed by a final extension at 72°C for 3 min. The same conditions were established for a second round of amplification with an annealing temperature increased to 62°C (Inácio et al., 2019). Previously known positive *B. vogeli* samples and negative samples were used as controls for the PCR.

Amplification products were separated on a 1% agarose gel stained with SYBR^©^ Safe DNA gel stain (Invitrogen, USA) and observed under UV light (Digidoc-It® Imaging System UVP, Canada).

### Sequencing and genetic analysis

The PCR amplicon positive for 18S rRNA were purified and sequenced by MACROGEN (Seoul, Korea).

The DNA sequences were compared to other sequences deposited in the GenBank (Benson et al., 2003) of the National Center for Biotechnology Information - NCBI (National Library of Medicine, 2024) using –the BLASTn algorithm (Altschul et al., 1990), where the sequences with an identity percentage >90% were considered as positive identification.

A phylogenetic relationships analysis was likewise performed using the 18S rRNA gene sequences obtained in the present study and the sequences available in GenBank: *B. vogeli* - Paraguay (MH100704.1; MH100702.1), *B. vogeli -* Italy (AY07925.1), *B. vogeli -* Taiwan (HQ148663.1), *B. vogeli -* China (HM590440.1), *B. microti -* Singapore (MK609547.1), *B. microti* - Congo (AB190459.1), *B. gibsoni* - Serbia (KJ696716.1), *B. conradae* - China (MH143389.1), *B. conradae* - USA (AF158702.1), *B. canis rossi* - Sudan (DQ111760.1), *B. rossi* - China (MH143395.1), *B. canis* - Estonia (KT008057.1), *B. canis* - China (MK256974.1) and *Hepatozoon canis -* Hungary (KF322143.1) were used as an outgroup. First, a multiple sequence alignment was generated using the ClustalW algorithm, then, the distance-based tree reconstruction was performed using the Maximum likelihood estimation, the Tamura 3-parameter (T92), bootstrapping 1,000 and the gamma distribution with invariant sites (G+I) model. The model was selected using the Akaike Information Criterion corrected (AICc) along with the Bayesian Information Criterion (BIC), and the MEGA X software was also employed (Kumar et al., 2018).

### Hematological analysis

Complete Blood Counts (CBS) were performed using the HumanCount 30^TS^ hematology analyzer (Human, Germany). The hematological parameters evaluated herein were: total erythrocytes and leukocytes, hematocrit, hemoglobin, Mean Corpuscular Volume (MCV), Mean Corpuscular Hemoglobin (MCH), Mean Corpuscular Hemoglobin Concentration (MCHC) and platelet count.

Thin blood smears were stained with modified May Grümwald-Giemsa (Biopack^®^, Argentina) and examined under the microscope for the cytological identification of hemoparasites, while the differential leukocyte count was determined by manual 100 cell counting in order to confirm the platelet count as well as to determine the presence of platelet aggregation. The reference values adopted were those recommended by Wittwer (2021).

Thrombocytopenia was classified according to Furlanello et al. (2005), as slight (142-100 x 10^9^ plateles/L), mild (99-50 x 10^9^ plateles/L), moderate (49-25 x 10^9^ plateles/L) and severe (< 25 x 10^9^ plateles/L).

### Statistical analysis

All data obtained by the hemograms was analyzed using descriptive statistics. The normal distribution was evaluated using the Shapiro-Wilk normality test (Shapiro & Wilk, 1965). The Mann-Whitney U-test two-tailed analysis (Mann & Whitney, 1947) (p<0.05 was considered statistically significant) was used to compare hematological parameters between positive and negative samples. Statistical analysis was performed using PAST 4.15 software (Hammer et al., 2001).

## Results

**Figure 1:**
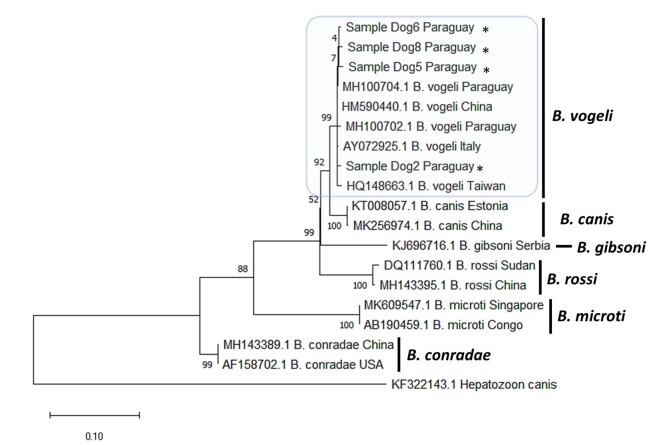
Phylogenetic relationships within the *Babesia* sp. based on 18S rRNA gene sequences. Percentages in nodes are supported in bootstrap values. Sequences obtained in this research (*).

Among the 35 samples analyzed in this study, only the 11% (4/35) were found positive for piroplasm confirmed by PCR targeting the 18S rRNA gene. Genetic sequencing analysis revealed that the piroplasm species was the *B. vogeli*, with an identity percentage between 99-100% with the reference sequence available in the GenBank. Phylogenetic analysis based on 18S rRNA gene indicated positive samples sequences clustered with *B. vogeli* sequences reported from Paraguay (MH100704.1, MH100702.1), China (HM590440.1), Italy (AY072925.1) and Taiwan (HQ148663.1), confirming that the piroplasm detected in these samples corresponded exclusively to *B. vogeli* (Figure 1). The sequences obtained in the present study were deposited in the GenBank NCBI (Accession Nº.: PV154041, PV154147, PV154163, PV155115).

**Figure 2:**
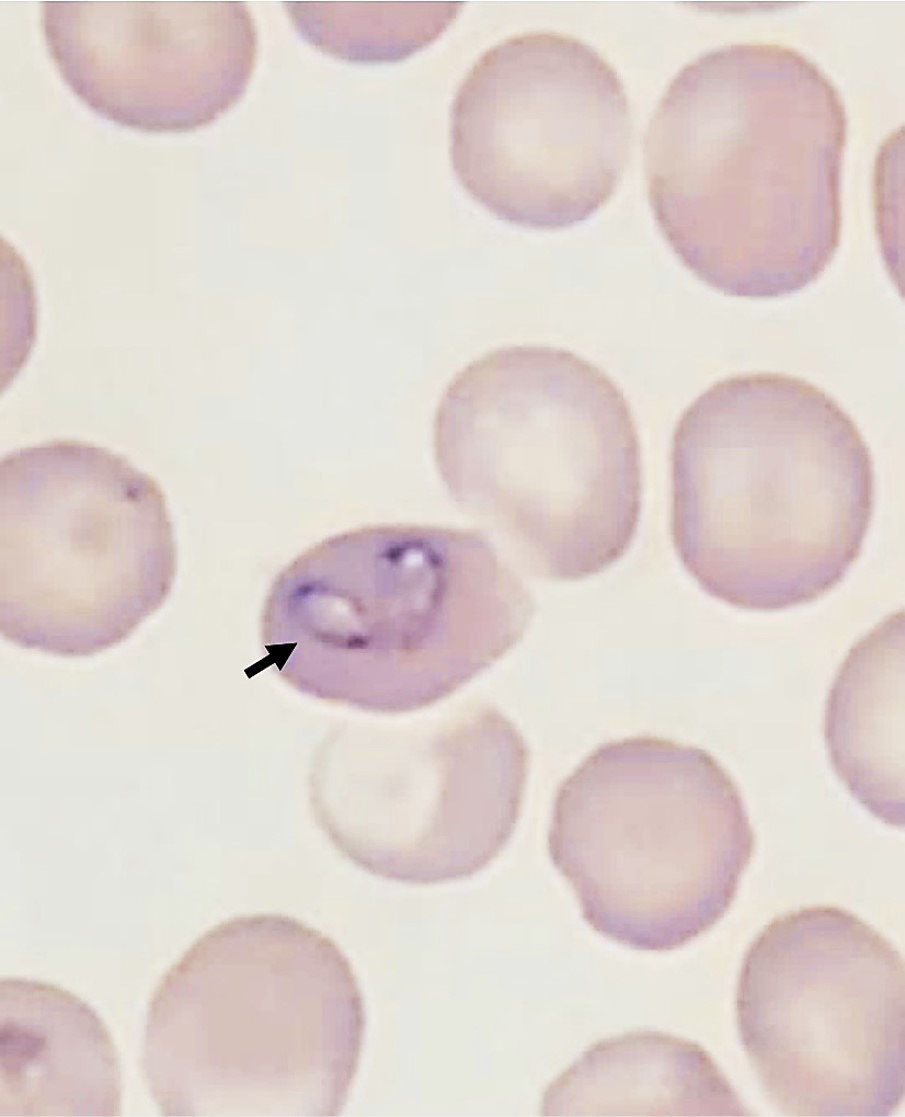
Peripheral blood smears from dogs naturally infected indicating the piroplasm inside the erythrocyte. Immersion microscopy (1000 x). Stained using May Grünwald-Giemsa.

Among the total samples analyzed, only two samples were found to be positive by microscopic observation of the *Babesia* sp. in the erythrocytes (Figure 2), being subsequently tested by PCR and sequenced, resulting in the *B. vogeli* findings. Although the presence of intraerythrocytic piroplasm was not microscopically observed in two samples, they were positive by sequencing.

In relation to the variable sex, breed and age, most of the blood samples 75% positive (3/4) with *B. vogeli* were of female, mixed breed and adult dogs (Table 1).

**Table 1:** Clinical parameters and relevant laboratory alterations of the dogs infected with *Babesia vogeli.*

**Sample ID**	**Variables demographic**			**Clinical signs**	**Hematological abnormalities**	**Blood smear**	**PCR results 18S rRNA and sequencing**
	**Breed**	**Age (years)**	**Sex**				
1	Mixed-breed	2	Female	Unknown.	Mild thrombocytopenia.	Intraerythrocytic large, pear-shaped forms morphologically compatible with *Babesia* sp.	*B. vogeli*
2	Mixed-breed	0,11	Female	Unknown.	Slight thrombocytopenia, severe normocytic normochromic no regenerative anemia leukopenia, neutropenia, lymphopenia, eosinopenia.	Intraerythrocytic large, pear-shaped forms morphologically compatible with *Babesia* sp.	*B. vogeli*
3	Weimaraner	7	Female	Pale and icterus mucous membranes, anorexia, apathy.	Slight thrombocytopenia, severe normocytic normochromic, leukocytosis, neutrofilia, lymphocytosis, eosinopenia, monocytosis.	Not observed	*B. vogeli*
4	Mixed-breed	3	Male	Depigmented areas around the eyes and noses.	Slight thrombocytopenia, monocytosis.	Not observed	*B. vogeli*

ID: Identification; PCR: Polymerase Chain Reaction

Of the hematological parameters analyzed herein, no significant differences were observed between *B. vogeli* positive and negative dogs (Table 2). The clinical signs were described in only two dogs, since the other two dogs were outpatients and only their samples were submitted for laboratory analysis. The clinical signs reported in the two dogs (50%) infected with *B. vogeli* were pale and icteric mucous membranes, anorexia, apathy, as well as depigmentation around the eyes and noses.

**Table 2:** Hematological parameters in dogs positive and negative for *Babesia vogeli.*

**Parameters**	**Positive**		**Negative**		***P-value***	Reference range*
	**Mean±SD**	**Median**	**Mean±SD**	**Median**		
RBC (x 10^12^ /L)	4.09±2.88	3.99	4.23±1.63	4.47	0.938	5.5-8.5
HCT (%)	27±18.35	27	27±9.51	28	0.897	37-50
HGB (g/L)	96.3±65.68	95.5	92.9±32.39	95	0.836	120-180
MCV (fL)	66 ±2.38	66	64±5.71	65	0.639	60-77
MCHC (g/L)	347.5±18.93	355	345.2±14.35	350	0.419	300-350
MCH (pg)	23±1.89	24	22±2.09	22	0.429	20-25
WBC (x 10^9^/L)	12.4±8.41	10.85	15.34±13.61	12.3	0.795	6-14
NEU (x 10^9^/L)	7.95±5.68	6.57	10.62±9.88	8.26	0.622	3.3-10
LYM (x 10^9^/L)	2.94±2.57	2.09	2.62±2.15	1.88	0.776	1-4.5
EOS (x 10^9^/L)	0.44±0.66	0.19	0.39±0.54	0.18	0.936	0.1-1.5
MONO (x 10^9^/L)	0.79±0.57	0.76	1.19±2.39	0.76	0.959	0.1-0.7
BAS (x 10^9^/L)	0	0	0.007±0.004	0	0.788	0-0.2
PLT (x 10^9^/L)	100±30.94	90	245.24±195.21	173	0.139	150-400

* Reference values were obtained from Wittwer (2021).

SD: Standard Deviation; RBC: Red Blood Cells; HCT: Hematocrit; HGB: Hemoglobin; MCV: Mean Corpuscular Volume; MCHC: Mean Corpuscular Hemoglobin Concentration; MCH: Mean Cell Hemoglobin; WBC: White Blood Cells; NEU: Neutrophils; LYM: Lymphocytes; EOS: Eosinophils; MONO: Mococytes; BAS: Basophils; PLT: Platelets.

Table 3 lists the hematological findings in dogs infected by *B. vogeli.* Hematological examinations revealed normocytic and normochromic anemia, by the association with moderate anisocytosis and polychromasia in dogs 2 as well as 3 (50%), additionally, both dogs presented eosinopenia. In dogs 1 and 4, the MCHM was above the normal values. Leukopenia, neutropenia and lymphopenia were found in dog 2, while dog 3 exhibited leukocytosis, neutrophilia and lymphocytosis. Monocytosis was registered in dogs 3 and 4. Thrombocytopenia was present in 100% of the positive animals.

**Table 3:** Hematologic findings of four dogs infected with *Babesia vogeli.*

**Parameter**	**Dog 1**	**Dog 2**	**Dog 3**	**Dog 4**	Reference range*
RBC (x 10^12^/ L)	7.2	**1.2**	**2.1**	5.9	5.5-8.5
HCT (%)	46	**8**	**15**	39	37-50
HGB (g/L)	164	**30**	**51**	140	120-180
MCV (fL)	64	63	68	67	60-77
MCHC (g/L)	**360**	320	350	**360**	300-350
MCH (pg)	23	20	24	24	20-25
WBC (x 10^9^/ L)	9.3	**4**	**23.9**	12.4	6-14
NEU (x 10^9^/ L)	4.8	**2.9**	**15.8**	8.3	3.3-10
LYM (x 10^9^/ L)	1.9	**0.9**	**6.7**	2.2	1-4.5
EOS (x 10^9^/ L)	1.4	**0**	**0**	0.4	0.1-1.5
MONO (x 10^9^/ L)	0.6	0.2	**1.0**	**1.5**	0.1-0.7
BAS (x 10^9^/ L)	0	0	0	0	0-0.2
PLT (x 10^9^/ L)	**145**	**76**	**96**	**84**	150-400

* Reference values were obtained from Wittwer (2021).

Values below the reference range are shown in bold font. RBC: Red Blood Cells; HCT: Hematocrit; HGB: Hemoglobin; MCV: Mean Corpuscular Volume; MCHC: Mean Corpuscular Hemoglobin Concentration; MCH: Mean Cell Hemoglobin; WBC: White Blood Cells; NEU: Neutrophils; LYM: Lymphocytes; EOS: Eosinophils; MONO: Mococytes; BAS: Basophils; PLT: Platelets.

## Discussion

Overall, *B. vogeli* has been the most frequently detected species in dogs in South America (Assad et al., 2020; Di Cataldo et al., 2020; Galván et al., 2018; Inácio et al., 2019; Silva Barbosa et al., 2020). The results herein showed a low prevalence of the *B. vogeli* compared to those detected in Brazil by Santos et al. (2020) and Silva Barbosa et al. (2020), which varied between 77.4-18.5%, respectively. The observed differences may be attributed to the type of samples analyzed in these studies, as they only included blood samples from dogs that tested positive for *Babesia* sp. by microscopic observation, whereas in the present study, molecular techniques were applied to all sampled dogs regardless of their microscopy results.

However, the results herein were higher than those reported in Colombia (Vargas-Hernández et al., 2012), in Perú (Cerro Temoche et al., 2018), in Brasil (Assad et al., 2020), in a previous study from Paraguay (Inácio et al., 2019), in Argentina (Eiras et al., 2008) and in Chile (Di Cataldo et al., 2020), with 1.1, 1.4, 3.6, 5.4, 5.9 and 6.2% of positives samples, respectively. This could be due to differences in the primers used, the sample size and the sampling season, considering that in the present research, sample collection was mainly performed in spring, given that the temperature in the referred season favors the reproduction of the insect vector. Dantas-Torres (2015), states that the spring and autumn are becoming longer seasons, along with a warmer winter season, which may contribute to the increasing distribution of several tick species.

Piroplasms are rarely found in the bloodstream, but they may be more frequently present in the acute phase of infection, where the parasitemia is detectable (Carini, 1948; Carini & Maciel, 1914; Loretti & Barros, 2004; Silva et al., 2011). Conversely, Vargas-Hernández et al. (2012), reported the presence of piroplasm in blood smears associated with positive PCR results, indicating that the animals were in the acute phase of the disease. Therefore, based on what has been described, it could be assumed that dogs 1 and 2 of this research were in the acute phase, while dogs 3 and 4 were in the subclinical or chronic phase of the disease, and that this could generate discordant results between the microscopic observation and the PCR.

Furthermore, Solano-Gallego et al. (2016) mentioned that the microscopic evaluation sensitivity is lower when compared to the molecular diagnosis. While the different forms of *Babesia* can be distinguished by blood smear, light microscopy is very specific and can be used to diagnose most dogs infected with the larger forms (*B. canis*) (Solano-Gallego et al., 2008; Solano-Gallego & Baneth, 2011), however, in the case of *B. vogeli* infections, it is less frequently detected in blood smears. For this infection, PCR-based molecular methods, which are more sensitive techniques, are more appropriate (Solano-Gallego et al., 2008).

Santos et al. (2020) and Davitkov et al. (2015), indicated that the highest cases of *Babesia* sp. were reported in purebred male dogs. Meanwhile, Silva Barbosa et al. (2020) and Araujo et al. (2015) recorded higher cases in female and mixed breeds. There is much disparity in the results in relation to sex and breed variables in different investigations, may be due to non-statistical associations with *Babesia* sp. infection with such factors (Castro et al., 2020). In terms of age, a higher number of cases was observed in young dogs (Davitkov et al., 2015; Silva Barbosa et al., 2012, 2020). Young animals are often more susceptible to infections because their immune system may not yet be fully developed (Silva Barbosa et al., 2020).

The clinical manifestations of canine babesiosis reported in this study were similar to those described in other studies: apathy, anorexia, fever, lethargy, fatigue, diarrhea, brownish-red or pale mucous membranes, jaundice, splenomegaly, vomiting, modified faeces and renal disease (Davitkov et al., 2015; Irwin, 2010; Máthé et al., 2006; Matijatko et al., 2012; Pawełczyk et al., 2022).

Similar results concerning the decrease in red blood cells were described by Assad et al. (2020), with 67% of dogs presenting anemia associated with anysocitosis and polychromasia. Likewise, Solano-Gallego et al. (2008) and Furlanello et al. (2005), established that 93.1 and 74% of *Babesia*-infected dogs had normocytic and normochromic anemia, respectively.

Regarding the MCHC, Zygner et al. (2007), reported 21% of dogs with values above the reference range, suggesting anemia caused by intravascular hemolysis, however, *in vitro* hemolysis could be the cause of this increase in some cases (Brockus & Andreasen, 2003). In this context, this could explain the MCHM increase found in the present work, considering that anemia was not observed, thus, the increase in MCHC could be related to an inadequate handling of the sample in the pre-analytical stage.

The alterations in the leukogram were very variable among the different investigations, with the presence of leukopenia, neutropenia, lymphopenia, leukocytosis, monocytosis and eosinophilia (Assad et al., 2020; Furlanello et al., 2005; Zygner et al., 2007). Considering the classification proposed by Furlanello et al. (2005), 75% (dogs 2, 3 and 4) had a mild thrombocytopenia and 25% (dog 1), a slight thrombocytopenia. Thrombocytopenia was the hematological alteration observed in all samples in this study. These results are consistent with the findings of others studies, such as the works from Vilela et al. (2013), Santos et al. (2020), Rawangchue and Sungpradit (2020) or Salem and Farag (2014), reporting 81.1, 95.8, 96.9 and 100% of thrombocytopenia, respectively. The mechanism of platelet decrease in the *Babesia* infection is still poorly understood (Furlanello et al., 2005). The isolated occurrence of thrombocytopenia, without alterations in other hemogram parameters, has been described in many cases of babesiosis and could be related to the immune system, splenic sequestration, platelet destruction, peripheral platelet consumption due to hemolytic alteration or vascular injury, increased body temperature and disseminated intravascular coagulation (Barić Rafaj et al., 2013; Wilkerson et al., 2001).

The quantitative results obtained are generally contradictory, ranging from the absence of anemia to severe anemia, neutropenia or leukocytosis, among others. These conflicting results may be explained by the heterogeneity of the studied populations and the time of sampling variable (Assad et al., 2020). Nevertheless, the first alterations are usually thrombocytopenia, eosinopenia and lymphopenia (Bourdoiseau, 2006). In addition, Solano-Gallego et al. (2008) studied dogs with *B. vogeli* infection and observed that they did not show a homogeneous clinicopathological pattern.

The main limitations of the present work were the number of samples analyzed because of limited economic resources, moreover, given the lack of technology, it was not possible to perform the quantitative PCR for all PCR-positive samples, taking into account that two of them were positive by PCR and negative by microscopic observation. Finally, the diagnostic method used to rule out other hemoparasites was established by blood smear observation, which is less sensitive and specific than molecular techniques.

Despite these limitations, the present study provides information on hematologic alterations in dogs with *Babesia vogeli.*

## Conclusions

The results of this study confirm the presence of *B. vogeli* in samples from dogs living in an urban area of Paraguay, presenting anemia and thrombocytopenia as the main hematological alterations.

Molecular techniques represent a very useful tool for the correct diagnosis of babesiosis, being used routinely at the clinical diagnosis process, given that this disease may often be underdiagnosed and confused with other pathogens transmitted by vectors that present similar hematological alterations. Therefore, accurately identifying the causative agent is crucial for administering the appropriate treatment.
